# Informed recruitment in partner studies of HIV transmission: an ethical issue in couples research

**DOI:** 10.1186/1472-6939-10-14

**Published:** 2009-08-27

**Authors:** Louise-Anne McNutt, Elisa J Gordon, Anneli Uusküla

**Affiliations:** 1 Department of Epidemiology and Biostatistics, University at Albany, State University of New York, Rensselaer, NY, USA; 2 School of Public Health, Tbilisi State Medical University, Tbilisi, Georgia; 3 Institute for Healthcare Studies, Division of Organ Transplantation, Department of Surgery, Feinberg School of Medicine, Northwestern University, Chicago, IL, USA; 4 Department of Public Health, University of Tartu, Ravila Tartu, Estonia

## Abstract

**Background:**

Much attention has been devoted to ethical issues related to randomized controlled trials for HIV treatment and prevention. However, there has been less discussion of ethical issues surrounding families involved in observational studies of HIV transmission. This paper describes the process of ethical deliberation about how best to obtain informed consent from sex partners of injection drug users (IDUs) tested for HIV, within a recent HIV study in Eastern Europe. The study aimed to assess the amount of HIV serodiscordance among IDUs and their sexual partners, identify barriers to harm reduction, and explore ways to optimize intervention programs. Including IDUs, either HIV-positive or at high risk for HIV, and their sexual partners would help to gain a more complete understanding of barriers to and opportunities for intervention.

**Discussion:**

This paper focuses on the ethical dilemma regarding informed recruitment: whether researchers should disclose to sexual partners of IDUs that they were recruited because their partner injects drugs (i.e., their heightened risk for HIV). Disclosing risks to partners upholds the ethical value of respect for persons through informed consent. However, disclosure compromises the IDU's confidentiality, and potentially, the scientific validity of the research. Following a brief literature review, we summarize the researchers' systematic evaluation of this issue from ethical, scientific, and logistical perspectives. While the cultural context may be somewhat unique to Eastern Europe and Central Asia, the issues raised and solutions proposed here inform epidemiological research designs and their underlying ethical tensions.

**Summary:**

We present ethical arguments in favor of disclosure, discuss how cultural context shapes the ethical issues, and recommend refinement of guidance for couples research of communicable diseases to assist investigators encountering these ethical issues in the future.

## Background

An estimated 33 million people are infected with HIV worldwide, including about 2.5 million newly infected in the past year. [1] Efforts to curb HIV transmission address a host of social, cultural, and clinical issues through important and diverse research studies to improve prevention and intervention methods. While much attention has been paid to the research ethics surrounding clinical trials of HIV treatment and prevention, [2-20] less discussion has focused on observational studies of HIV transmission involving sex partners. Couples research is crucial to intervention development and raises unique ethical dilemmas that are not specifically addressed by regulations, laws, and ethical standards [2-4,21-23].

In couples research, the primary ethical tension entails balancing the risks and benefits to the participating individuals, to the couple, and to society. Complicating the issue, the investigators' ability to conduct scientifically valid research may be inversely related to their ability to fully protect the well-being of individual participants. For example, in studies of sexual transmission of HIV among injection drug users (IDU) and their partners, we typically recruit IDUs and ask that they recruit their sexual partners. Informing sexual partners that they are eligible for the study because the partner is an IDU – a group with high HIV prevalence – may immediately prompt them to change their risk behaviors or leave the relationship thereby biasing estimates of transmission rates. The disclosure of risks to sexual partners can also be justified as a means of minimizing harms to others. Yet not informing the partners may result in investigators watching the natural course of disease transmission in an uncontrolled environment. Couples research thus raises important questions: What are researchers' obligations to disclose information about HIV risk when HIV risk is a central component of study selection and eligibility? How much information should be provided to potential recruits about their risk as part of the informed consent process? How do researchers balance the rights of each individual within a couple, and study validity, in the informed consent process?

This paper focuses on ethical dilemmas that arise when IDUs are recruited into behavioral research on HIV that will involve their sexual partners. Should researchers disclose to these partners that they were recruited because their partner injects drugs (i.e., their heightened risk for HIV)? Disclosing would uphold the ethical value of respect for persons and serves to minimize harm to them. However, in research involving couples, disclosure compromises the IDU's confidentiality. Investigators may have conflicting obligations to members of the couple, such as in this study: there is a conflict between protecting the interests in confidentiality of the IDUs recruited for the study and protecting the interests of their sexual partners – some of whom may not know their partner is an IDU – in being able to make informed choices. Disclosure also potentially weakens the scientific validity of the research if it causes some IDUs not to enroll because they do not want this disclosure to be made. Following a brief literature review, we summarize the researchers' systematic evaluation of this issue from ethical, scientific, and logistical perspectives. While the cultural context may be somewhat unique to Eastern Europe and Central Asia, the issues raised and solutions proposed here inform epidemiological research designs and their underlying ethical tensions.

The tension between confidentiality and informed recruitment in HIV transmission research first received considerable attention with the publication of a Ugandan cohort study conducted between 1994 and 1998. [24-28] Researchers identified 415 HIV-discordant couples from a large randomized trial designed to study the impact of bacterial sexually transmitted diseases on HIV transmission. [24] The couples were then followed to identify factors associated with HIV transmission. Substantial harm reduction education, free condoms, and voluntary, confidential HIV testing and counseling were offered to all participants. However, researchers were legally obligated not to inform participants who were HIV-negative about their partners' HIV-positive status. Debate ensued about the choice of confidentiality versus disclosure of partner HIV status. [25-28] A decade later the debate continues with no clear resolution.

The issue is further complicated by the legal issues that surround HIV. In much of the world, including some US states [29] and European countries, [30] disclosure by a physician of an individual's HIV status without the patient's consent is illegal. In other locations, physicians have a duty to warn partners through mandated notification programs.[31] Further, professional ethics codes may differ from local laws, complicating these issues.[32] Lastly, laws pertaining to medical care and observational research conducted outside medical settings may differ.

Informed consent of research study participants begins with the recruitment process. Typically, recruiting couples starts with recruitment of the "index-participant" – individuals either infected with HIV or at very high risk for HIV (e.g., IDU). The sexual partner is commonly recruited with the assistance of the index-participant. The extent of information disclosed to sex partners as to why they are being sought for study inclusion remains controversial. Some studies involve specific information about eligibility related to substantial HIV risk while others do not. Major initiatives, such as the HIV Prevention Trial Network (HPTN), have developed formal guidelines that address fundamental research ethics issues, but these do not prescribe a specific approach for "informed selection."[2] Providing accurate HIV risk information during the informed consent process to all participants is consistent with the HPTN guidelines (Sugarman J, personal communication, 2008). The UN AIDS guidance for HIV prevention trials also highlights that individuals be told the specific reason for study eligibility. [4]

### HIV in Estonia

In Estonia, the primary reservoir for HIV is the IDU community. By the turn of the century, this small Baltic state had the highest HIV incidence in Europe.[33,34] Similar to nearby cities in Russia, the IDU community was the epicenter of the epidemic, and HIV spread quickly during the post-independence transition period of the late 1990s.[34,35] About half of all IDUs in Estonia are infected with HIV and over 90% are infected with hepatitis C virus (HCV). [36-38] The transmission of HIV between IDUs is well understood and some intervention programs are available, including free voluntary counseling and testing, needle exchange programs and drug treatment. [39-50] However, improved HIV prevention and intervention programs are critically needed to curb the spread of HIV among IDUs in general, and between IDUs and their sexual partners. [39,40,45,51-54]

The primary mode of HIV transmission from the IDU community to the general population is thought to be through sexual contact.[45] However, insufficient research has focused on improving interventions to interrupt sexual transmission between IDUs and their partners. Because the IDU community is a substantial reservoir for HIV in Eastern Europe and Central Asia, understanding and stopping transmission from this group is essential to slow the spread of the epidemic. These studies must be conducted against the backdrop of a changing social structure in the region, largely related to major changes in the political and economic environment following independence.

## Discussion

### The Estonian Study

The study discussed here, a joint project of Estonian and US researchers, took place in 2007 following Institutional Review Board approval for the study granted by both US and Estonian institutions. The study objectives were threefold: 1) assess the amount of HIV serodiscordance among IDUs and their sexual partners, 2) identify barriers to harm reduction, and 3) explore ways to optimize intervention programs. Thus, we wanted to include IDUs, either HIV-positive or at high risk for HIV, and their sexual partners to gain a more complete understanding of the barriers to and opportunities for intervention.

Injection drug users were recruited into research through harm reduction programs, at known locations of drug use, and by using respondent-driven sampling.[55,56] Once an IDU was recruited, he/she was asked to invite both a sexual partner(s) who does not use injection drugs and other IDUs to participate in the study. Index-participants were given two sets of coded cards to hand out (one set for sexual partners and the other for IDUs as part of respondent-driven sampling). Each individual was provided a written informed consent form after the researcher orally disclosed the purpose of the study, procedures, risks and benefits of participation, participants' rights, and provided an opportunity to read the form (containing the same information on the study) and ask questions. Each participant was interviewed separately and blood was obtained for HIV and HCV testing. The survey tool collected detailed information about drug use, sexual behaviors, HIV testing, knowledge about HIV transmission, and attitudes about HIV. Anonymous codes linked records. (Anonymity was important because IDU is a crime in Estonia [57,58] and the equivalent of a US Certificate of Confidentiality [59,60] does not exist to protect participant data.) All participants received counseling as part of the HIV testing and the counseling program provided in Estonia. All participants were asked to return for test results. Every aspect of the study was reviewed multiple times by the investigators to ensure IDUs received adequate protection.

### Informed Consent Process

During the informed consent process, research staff took great care to be transparent regarding all aspects of the research study. All participants were told we were specifically studying HIV, including it's potential to be sexually transmitted, and provided counseling around HIV testing. We explained to IDUs the reason for recruitment and all relevant study procedures. But it was less clear what to disclose to the recruited sex partners. To fully inform sexual partners of the study's purpose and specific reason for their study eligibility might require a disclosure of the index-participants' drug use and heightened risk of HIV.

Thus, the crux of the ethical dilemma was the informed consent process for the *sexual partners*, and specifically the explanations about partner selection and the study purpose. Our team generated multiple conceptual options for informed consent statements, with the varying levels of detail in italics for comparison:

1. "You were selected to participate in this study on *relationships and risk behaviors *because your partner volunteered for the study and was asked to invite his/her sex partner."

2. "You were selected to participate in this study of *injection drug users *and their sex partners because your partner volunteered for the study and was asked to invite his/her sex partner."

3. "You were selected to participate in this study of *injection drug users *and their sex partners because your partner volunteered for this study and was asked to invite his/her sex partner. *About half of injection drug users are infected with HIV and we are particularly interested in understanding risk behaviors of injection drug users who are infected with HIV and their partners. In particular, we want to learn what behaviors result in the highest risk of HIV transmission and better ways to reduce risk of transmission*."

The first statement provides no information to the partners recruited for the study about their risk of infection now or in the near future. The second statement differs from the first by specifying that the partner is an IDU. This information is especially revealing because it is common knowledge among residents of Estonia that many IDUs are HIV positive. The third statement clearly states partners' potential risk of HIV if they are not actively preventing transmission (e.g., using condoms).

The research team was unable to use the third statement, due to concerns of the local ethics committee about such specific, yet ambiguous communication that could further stigmatize IDUs. While it may appear arbitrary that one stigma (i.e., IDU status) can be shared while another (i.e., HIV status) can not, it is not uncommon that inconsistencies in sensitivity about exposures exist in many countries. The research team debated the first two options at length. The decision would have health, scientific, and ethical consequences. In the following we describe the process we underwent and factors that informed our decision.

### The Process of Ethical Deliberation

Investigators originally planned to use the first statement, that is, to not disclose the study's primary focus on IDUs and HIV transmission risk to their sexual partners. Driven by our prior experience with vulnerable populations, we designed the study to fully protect IDUs while maximizing their comfort with participating in the research project. However, further reflection on the public health and ethical implications of this approach to disclosure led the research team to reconsider. We sought input from multiple sources for over a year prior to study implementation, including informal conversations with IDUs, NGOs (e.g., needle exchange programs), researchers, ethicists, friends and family members of IDUs, and others

As the discussion unfolded, substantial concern focused on the asymmetrical knowledge between investigators and study participants. The researchers would know more about the risk of continued unprotected intercourse with the index-participant (e.g., discordant HIV status, rare or no use of condoms) than the partner. Proponents of partner notification advocate a defined legal limit on confidentiality of the physician-patient relationship, the value of providing life-saving treatment for infected partners, and overall benefit to the public's health. Challengers to partner notification question whether mandatory partner notification laws for HIV will have an overall negative impact on both medical care and public health due to the loss of patients' ability to trust their physicians with sensitive information. [61-64] Investigators must consider many factors when developing a research study that figure into designing the process of informed consent. The key factors that arose in our research deliberations included protection of vulnerable populations, confidentiality waivers, validity of scientific results, differential risk for partners based on informed consent, and research logistics, all of which are discussed below.

### Protection of Vulnerable Populations

In Estonia, research ethics committees consider IDUs among the vulnerable potential study participants. Known IDUs are marginalized for several reasons, including: a) their (illegal) behavior, b) most are members of an ethnic minority (about 80% are Russian-speaking), c) many have served time in jail or prison, d) many are poor, and e) about half are known to be HIV positive [34]

Sex partners of IDUs, who are not themselves IDUs, typically share only two of these vulnerabilities: Russian-speaking minority and low socioeconomic status. Another important factor to consider related to vulnerability is that divorce in Estonia is not uncommon. [65] As partners learn of the index-participant's HIV/IDU status, they may consider the option of divorce. Divorce is a socially acceptable option, and viable since sufficient housing exists in Estonia. By contrast, leaving a sexual partner is not a viable cultural or economic option in many other countries.

### Confidentiality Waiver

For the IDU participants, requiring disclosure about drug use may result in feelings of vulnerability or frustration if they want to participate but do not want disclosure. Ensuring the confidentiality of study participants is a paramount human subjects projection. However, other equally pressing human subjects protections – informing partners of why they are being recruited into the study, must be balanced against protecting the confidentiality of the IDU. Accordingly, research participants commonly provide limited waivers of confidentiality for many studies. These waivers may be for protected information to be provided to researchers (e.g., medical records) or disclosed among research participants (e.g., research on couples or family counseling). In these situations, participants typically provide waivers of confidentiality that are limited to requirements for good scientific research. Indeed, in our study the index-participant willingly disclosed their sexual relationship with the referred partner(s).

Is there an ethical difference between requiring information disclosure about a person's drug use or HIV infection compared to disclosing information about a person's sexual relationship in order to participate in the study? Disclosure of IDU in Estonia to a partner could present significant harms to the IDU. In Estonia, not only is IDU illegal, but it is also illegal for a person who knows he/she is HIV-positive to transmit HIV to a person who was not aware of this risk, and criminal convictions have occurred. [66]

Conversely, waiving limited confidentiality is commonly required in research. If the requirements for research participation are unacceptable, then individuals can choose not to participate. In Estonia, the same HIV testing is free and available as is the counseling provided to study participants, which is comparable to that provided in the wider community. Thus, there is no overly compelling benefit for individuals to participate. The study's modest compensation was attractive to community members but not excessive such that a typical low-income IDU would unduly agree to unacceptable conditions. (This assessment was validated by the fact that some IDUs did not participate and others did not refer partners.)

### Validity of Research Findings

For a research study to be ethical it must be scientifically valid. No risk is warranted if the study cannot be informative. From an epidemiologic perspective, the greatest risk to generalizability regarding informed recruitment is selection bias. If index-participants know that their sex partner(s) will be told about their drug use, then only partners who already know about this risk behavior will likely be referred for participation. Limitations on study participation driven by the consent process would, in turn, limit the effectiveness of resultant interventions to those with characteristics similar to the group studied. While our study was cross-sectional, the same issue applies to longitudinal studies designed to estimate HIV transmission rates more broadly.

Based on interviews with IDUs, we hypothesize that drug users who do not want their partners to know about their drug use are unlikely to recruit them for a study on HIV risk and transmission. It is not only the researchers who the IDU must trust, but also the other individuals the partner may encounter at the research location. Thus, the primary disadvantage of informed recruitment – selection bias – may likely be introduced by study participants, and that additional selection bias introduced by an in-depth informed consent is minimal. While empirical research is needed to formally test this hypothesis, it is supported by other researchers' experience working with IDUs (D. Des Jarlais, personal communication, 2008).

### Risk

Arguments against and in favor of disclosure of the index participant's IDU status can be made depending on different interpretations of risk of HIV transmission to the public's health. A key argument against disclosure is that research participation does not *increase *the risk of HIV transmission/infection by participants. Even if the sex partners were not informed of the IDU status of the index participants during the recruitment process, all participants received state-of-the art education about behavioral risk factors and harm reduction techniques, which should minimize their risk of transmission. Another argument is that providing additional risk information does not necessarily enhance the study participant's protection. Empirical research shows that these educational and behavioral risk reduction interventions minimally affect sexual behavior. [51-54] That is, many partners continue to undertake risky sexual relations with IDUs after being informed. Accordingly, if the study provides limited risk reduction benefit, why introduce the potential for selection bias that would jeopardize the results? Such empirical evidence may lead some to conclude that disclosure is not necessary. For these and other reasons, nondisclosure of the index participant's IDU status is still commonly practiced in the US and abroad.

Nevertheless, there are substantial arguments in favor of disclosure. First, informing partners, who are study participants, enables them to become aware of the urgency with which to undertake precautions. It is imperative to ensure that potential study participants make informed choices, even if how they choose to act thereafter may not be healthful. Thus, doing so minimizes harms to study participants. Another justification for disclosure may be drawn from interviews with the IDUs in Estonia. Many partners leave the relationship upon learning about drug use. Because HIV is substantially more prevalent in the IDU community, leaving a relationship with an IDU *does *reduce a partner's risk of HIV infection. Thus, from the partners' perspective, this is an effective method of risk reduction that they *can *take and many are *willing *to take.

Moreover, nondisclosure may pose the potential for emotional harm to partners and the broader community if it became known that researchers knew some HIV-negative participants were having unprotected sex with HIV-positive individuals. Partners may feel betrayed and the community may feel manipulated. Such feelings could easily transform into distrust and reluctance to participate in research in the future, as has occurred among African Americans in the US. [67,68]

### Logistics

Paper forms were used for most data collection, causing a delay in data entry. While the index-participant's *IDU *status was known, most partners were enrolled before information about HIV status was available. Nonetheless, studies of HIV transmission that include diverse risk groups and follow the transmission risk to multiple sex partners may benefit from rapid tests and real time data processing to provide appropriate information about disease risk to recruited individuals. Without these technologies, general statements related to risk of HIV can be made to all participants.

### Resolution

For our study, we could not add IDU information to the partner's informed consent form because of the legal concerns about revealing HIV status. Thus, our pragmatic compromise was to tell all index participants to only recruit sex partners who knew about their drug use.

### Summary

During the design phase of this study, we identified ethical tensions unique to the process of studying couples. From an epidemiological perspective, when the couple is of central interest then it is the unit of study and analysis. However, from a research ethics perspective, the individuals, the couple, and the greater community all are important to consider and warrant human subjects protections. At times what is best for one person in a couple may not be best for the other. For couples research pertaining to HIV sexual transmission, we posit that failing to fully inform partners about the rationale for inclusion in the study is ethically disconcerting. Intentionally limiting disclosure to sexual partners remains ethically problematic given the potential for continued risk that exists among HIV-discordant couples with unsafe sex behaviors. While institutional review boards have developed sensitivity to cultural appropriateness for research in developing countries, refinement of guidance for couples research of communicable diseases is needed to address ethical issues such as those raised herein.

We suggest that further guidance for both cross-sectional and longitudinal studies of HIV transmission is needed regarding informed recruitment. While in our example recruitment was anonymous and limited to IDUs and their sexual partners, in longitudinal HIV transmission studies researchers recruit diverse risk groups (e.g., IDU, commercial sex workers, MSM) and follow transmission for multiple waves. Thus, specific HIV risk (e.g., HIV status of sexual partners) often will not be known at the time of study interviews. We suggest that research participants can be accurately told that they were recruited for the study because they are connected to a group at higher risk for HIV then the general population. The detailed explanation of this risk may vary by wave of recruitment and by region. Additionally, in longitudinal studies, we suggest research participants be reminded that they were recruited for the study due to their high risk for becoming infected with HIV and harm reduction counseling be provided with each follow-up blood test.

## Conflicting interests

The authors declare that they have no competing interests.

## Authors' contributions

LAM and AU discussed the issues covered in the paper for over a year in preparation for the study and during the study. They asked EJG to join the discussion and provide input from a trained ethicist perspective. LAM and EJG drafted the manuscript based on these discussions and AU edited the manuscript in an iterative process.

## Pre-publication history

The pre-publication history for this paper can be accessed here:


